# The risk of malnutrition as a predictor of arrhythmia recurrence after catheter ablation in patients with paroxysmal non-valvular atrial Fibrillation and heart failure with preserved ejection fraction

**DOI:** 10.1371/journal.pone.0317721

**Published:** 2025-01-31

**Authors:** Zixi Zhang, Cancan Wang, Qiming Liu, Yichao Xiao, Jiabao Zhou, Keke Wu, Yunying Huang, Zeying Zhang, Shiping Liu, Qiuzhen Lin

**Affiliations:** 1 Department of Cardiology, The Second Xiangya Hospital, Central South University, Changsha City, Hunan Province, People’s Republic of China; 2 Department of Endocrinology, The Second Xiangya Hospital, Central South University, Changsha City, Hunan Province, People’s Republic of China; 3 Department of Nutritional Sciences, The Second Xiangya Hospital, Central South University, Changsha City, Hunan Province, People’s Republic of China; Tehran University of Medical Sciences, IRAN, ISLAMIC REPUBLIC OF

## Abstract

**Background:**

Malnutrition presents a significant challenge in managing patients with atrial fibrillation (AF) and heart failure with preserved ejection fraction (HFpEF), yet its impact on AF recurrence after catheter ablation in this population remains unclear.

**Methods:**

We conducted a retrospective analysis of 204 patients with paroxysmal non-valvular AF and HFpEF who underwent radiofrequency or cryoballoon ablation. Risk of malnutrition as assessed using three screening tools: the Controlling Nutritional Status (CONUT) score, Prognostic Nutritional Index (PNI), and Nutritional Risk Index (NRI)]. We examined the relationship between risk of malnutrition and AF recurrence post-ablation.

**Results:**

After a mean follow-up period of 11.2 ±  1.8 months, 43 patients (21.1%) experienced AF recurrence. Despite being classified as overweight or obese based on body mass index, many patients were at risk of malnutrition according to the CONUT score, NRI, and PNI. Adjusted analyses showed that higher CONUT scores (HR: 10.132; 95% CI: 2.545–40.336; *P* =  0.001), lower NRI (HR: 22.734; 95% CI: 6.399–80.776; *P* <  0.001), or lower PNI (HR: 9.469; 95% CI: 3.232–27.739; *P* <  0.001) were significantly associated with increased risk of AF recurrence. Restricted cubic spline regression revealed an inverted U-shaped relationship between the CONUT score and AF recurrence, and L-shaped relationships for both NRI and PNI with AF recurrence.

**Conclusions:**

Systematic nutritional assessment is crucial in patients with paroxysmal non-valvular AF and HFpEF. High CONUT scores, low NRI, or low PNI serve as independent predictors for AF recurrence. Further large-scale randomized controlled trials are required to validate these findings.

## 1. Introduction

Malnutrition is a major cause of mortality in developing countries. Notably, even in developed countries, approximately 20–50% of hospitalized patients experience malnutrition, and 70% exhibit a decline in nutritional status during hospitalization [1,2]. Recent studies have indicated a close association between malnutrition and the incidence of cardiovascular diseases [3,4], which is an issue currently receiving considerable attention. Atrial fibrillation (AF) and heart failure (HF) are prevalent cardiovascular diseases, particularly in patients with heart failure with preserved ejection fraction (HFpEF). HFpEF accounts for approximately 50% of all HF cases and is closely associated with AF [5,6]. The complex and interrelated mechanisms involved contribute to a poor long-term prognosis for these patients [7]. Existing evidence suggests that, compared to patients with heart failure with reduced ejection fraction (HFrEF), limited pharmacological interventions, such as sodium-glucose cotransporter 2 inhibitors, glucagon-like peptide-1 receptor agonists, and finerenone, may offer benefits to individuals with HFpEF [8–10]. Furthermore, for certain HFpEF patients with lower left ventricular ejection fraction (LVEF), angiotensin receptor-neprilysin inhibitors may reduce hospitalization rates [11]. In patients with both AF and HFpEF, appropriate management of AF can improve symptoms and clinical outcomes. These findings have prompted clinicians to explore novel non-pharmacological treatments, such as catheter ablation. While previous research has highlighted the predictive value of nutritional scoring in AF recurrence post-ablation [12], differences in underlying mechanisms between HFpEF and HFrEF may render existing evidence regarding the risk of malnutrition (i.e., nutritional risk) in patients with HFrEF inapplicable to those with HFpEF. Therefore, investigating the impact of malnutrition on AF recurrence in patients with AF and HFpEF holds considerable clinical relevance.

Current systems and scores for assessing nutritional risk primarily include both subjective and objective assessment tools. Objective assessment tools, such as the Controlling Nutritional Status (CONUT) score, Prognostic Nutritional Index (PNI), and Nutritional Risk Index (NRI), have been widely utilized in clinical settings for nutritional risk screening [13–15]. Existing research indicates that nutritional risk, as assessed by the CONUT score, NRI, or PNI, serves as an independent prognostic factor for adverse clinical outcomes in patients with acute coronary syndrome, peripheral arterial disease, HF or AF [16–20]. Additionally, a study by Zhu et al. [21] revealed that the CONUT score and NRI are optimal instruments for assessing the nutritional status of patients after AF ablation. Notably, patients with AF identified as susceptible to malnutrition exhibit an increased probability of recurrence. However, there is currently a lack of research regarding the nutritional status of patients with AF and HFpEF and the prognostic impact of nutritional risk on AF recurrence.

This study employed the CONUT score, NRI, and PNI to assess nutritional status in patients with paroxysmal non-valvular AF and HFpEF. Furthermore, we explored the potential influence of nutritional risk on the AF recurrence post-ablation.

## 2. Methods

### 2.1. Study inclusion criteria

**Fig 1:**
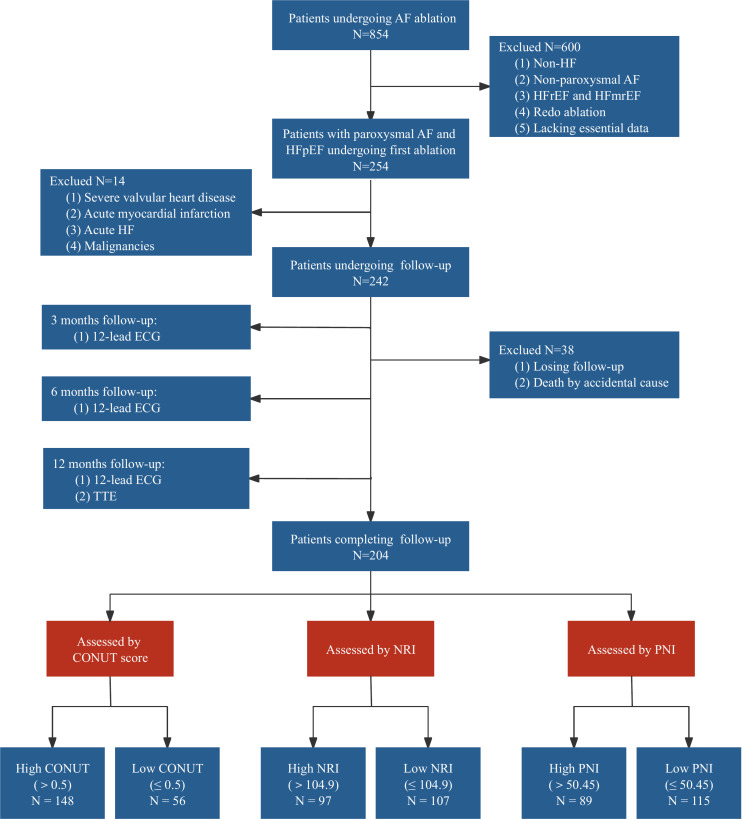
Study flowchart. AF, atrial fibrillation; CONUT, controlling nutritional status; ECG, electrocardiogram; HF, heart failure; HFmrEF, heart failure with mildly reduced ejection fraction; HFpEF, heart failure with preserved ejection fraction; HFrEF, heart failure with reduced ejection fraction; NRI, nutritional risk index; PNI, prognostic nutritional index; TTE, transthoracic echocardiography.

A retrospective cohort study was conducted, including 204 patients aged 18 years and older, diagnosed with paroxysmal non-valvular AF and HFpEF. These patients underwent either radiofrequency ablation (RFA) or cryoballoon ablation (CBA) for the first time at the Heart Intervention Center of the Second Xiangya Hospital of Central South University between January 2017 and December 2021. All patients underwent pulmonary vein isolation (PVI) and did not receive any additional linear ablation. The diagnosis of AF was confirmed by at least one 12-lead electrocardiogram (ECG) or a single-lead ECG lasting more than 30 seconds, further verified by a cardiac electrophysiologist. The diagnosis of HFpEF was established using the 2021 European Society of Cardiology diagnostic criteria, further confirmed using the H2FPEF score [22,23]. A H2FPEF score >  5 points confirmed the diagnosis of HFpEF. The exclusion criteria included AF triggered by hyperthyroidism, pregnancy, left atrial diameter >  50 mm, a history of cardiac surgery, severe valvular heart disease, acute myocardial infarction, acute HF, malignancies, and an inability to calculate the CONUT score, NRI, or PNI. Detailed information on the included studies can be found in Fig 1.

### 2.2. Clinical and laboratory data collection

Data were extracted by two investigators who were initially blinded to the hypotheses. The extracted data were subsequently cross-checked by a third investigator to verify the accuracy. Patient-related data will be made available only in deidentified form, in compliance with applicable data protection regulations. All clinical and laboratory data were collected through the electronic medical records system. Baseline information included demographic characteristics, fundamental details, comorbidities, laboratory results, transesophageal echocardiography (TEE) data, ablation procedures, medical interventions, and nutritional status. This study involving the collection and analysis of patient case data was conducted in accordance with ethical principles, and all procedures were carried out following the Declaration of Helsinki. The research protocol and data collection methods were assessed, and it was determined that formal ethics approval was not needed for this study. The study strictly adhered to patient confidentiality and privacy restrictions, and all the data were anonymized to ensure the protection of personal information.

### 2.3. Nutritional screening tools

The CONUT score was utilized as a screening tool for assessing the nutritional status of hospitalized patients. This scoring system incorporates serum albumin (ALB) concentration, total cholesterol (TC) concentration, and lymphocyte count (LC) [13]. The PNI was calculated using the formula: $10\times \;serum\;ALB\;\left(g/dL\right)+0.005\times LC\;\left(/{\text{mm}}^{3}\right)$ [14]. The NRI was calculated using the formula: $1.519\times serum\;ALB\;\left(g/L\right)+41.7\times (current\;weight\;in\;kilograms/ideal\;weight)$ [15]. The ideal body weight was determined using the Lorenz formula: $height\;\left(cm\right)-100-\left[\left(height\left(cm\right)-150\right)/4\right]$ for men and $height\;\left(cm\right)-100-\left[\left(height\left(cm\right)-150\right)/2.5\right]$ for women [24]. Body weight was measured using a calibrated electronic scale in kilograms, with patients wearing light clothing and no shoes. To align with previous research findings, the weight ratio of the current body weight to the ideal body weight was set to 1 when the current body weight exceeded the ideal body weight. Blood samples were collected using standard venipuncture techniques in the morning after an overnight fast. Serum was separated by centrifugation at 3000 rpm for 10 minutes and stored at –20°C until analysis. Serum ALB levels were measured using the colorimetric method based on the bromocresol green dye-binding assay. TC concentration was determined using an enzymatic colorimetric method. A complete blood count was performed using the Beckman Coulter automated hematology analyzer. Blood was collected in EDTA tubes and processed immediately for LC. Nutritional status for all patients was assessed using nutritional screening tools as shown in Table 1.

**Table 1:** Details of three nutritional screening tools.

Nutrition scores	Risk of malnutrition			
	Absent	Mild	Moderate	Severe
CONUT, points	0–1	2–4	5–8	9–12
ALB, g/dl	≥ 3.5	3.0–3.4	2.5–2.9	< 2.5
Score	0	2	4	6
TC, mg/dl	≥ 180	140–179	100–139	< 100
Score	0	1	2	3
LC, × 10^9^/L	≥ 1.60	1.20–1.59	0.80–1.19	< 0.80
Score	0	1	2	3
NRI, points	≥ 100	97.50–99.99	83.50–97.49	< 83.50
Formula	1.519 × ALB (g/L) + 41.7 × (weight in kg/ideal weight)			
PNI, points	≥ 38	–	35–38	< 35
Formula	10 × ALB (g/dL) + 0.005 × LC (/mm^3^)			

Abbreviations: ALB, albumin; CONUT, controlling nutritional status; kg, kilograms; LC, lymphocyte count; NRI, nutritional risk index; PNI, prognostic nutritional index; TC, total cholesterol.

### 2.4. Preprocedural management and ablation procedure

Preprocedural application of TEE and cardiac computed tomography angiography were employed to exclude left atrial thrombus and delineate pulmonary vein anatomy. If left atrial thrombus was detected, intensified anticoagulation therapy was initiated for a minimum duration of one month. Subsequent reassessment via TEE confirmed complete thrombus dissolution before considering catheter ablation. Comprehensive evaluation of stroke and bleeding risks was conducted using CHA_2_DS_2_-VASc and HAS-BLED scores. For patients on warfarin, the international normalized ratio (INR) was maintained between 2.0 and 2.5 before ablation, without discontinuing warfarin administration. Patients on novel oral anticoagulants (NOACs) discontinued these medications 12 hours before ablation. Additionally, management of concomitant comorbidities adhered to respective clinical guidelines.

All procedures were conducted under intravenous fentanyl sedation. Heparin was promptly administered post-interatrial septal puncture to maintain activated clotting time between 300–400 seconds. In the RFA procedure, the CARTO magnetic mapping system (Biosense Webster, Inc.) or Ensite three-dimensional mapping system (Saint Jude Medical, Inc.) was used to perform three-dimensional electroanatomic reconstruction of the left atrium. PVI was performed under the guidance of three-dimensional electrical reconstruction of the left atrium and digital subtraction angiography. In the CBA procedure, after bilateral pulmonary vein angiography, cryoballoon and Achieve mapping catheters (Medtronic, Inc.) were sequentially introduced into pulmonary veins for cryoablation. The Achieve mapping catheter was positioned at the ostium of bilateral pulmonary veins to assess the success of PVI. All procedures were performed by the same experienced electrophysiology team.

### 2.5. Postprocedural management and follow-up

Postoperatively, proton pump inhibitors were administered for at least 2 weeks to prevent esophageal injury. For patients receiving warfarin, anticoagulation therapy was resumed on the day of the procedure (maintaining INR between 2.0 and 3.0) once the patient showed no signs of hemorrhage. For those on NOACs, anticoagulation therapy could be resumed within one day and continued for at least 2 months. Antiarrhythmic drugs were continued for 3 months post-ablation, after which the cardiovascular specialist adjusted the treatment strategy based on the occurrence of AF recurrence.

Follow-up assessments were conducted via telephone or social media platforms such as WeChat at discharge, and at 3, 6, and 12 months post-ablation. A cardiac ultrasound was performed 12-month post-ablation. Additional examinations were recommended if patients presented with suspected symptoms related to arrhythmias. AF recurrence was defined as the occurrence of AF, atrial flutter, or atrial tachycardia lasting more than 30 seconds in a resting state, as recorded by a 12-lead ECG or wearable activity monitor after a 3-month blanking period following the ablation procedure.

### 2.6. Statistical analysis

Continuous variables are presented as the mean ±  SD or median (interquartile range) and were compared using Student’s t-test or the Mann–Whitney U test, as appropriate. Categorical variables, presented as n (%), were compared using the χ^2^ test or Fisher’s exact test. Receiver operating characteristic (ROC) curve analysis was performed to evaluate the sensitivity and specificity of the CONUT score, NRI and PNI, and to determine the cutoff values for predicting AF recurrence. The DeLong test was used to compare the area under the curve (AUC) of each nutritional screening tool [25]. The survival time distribution was evaluated by the Kaplan–Meier method, and the log-rank test was used for comparisons. A multivariate Cox proportional hazard model was constructed to compute hazard ratios (HRs), and a forest plot was used to investigate the associations between the three nutritional screening tools and AF recurrence. *P* values for trends were calculated using the quartile median value as a quasi-continuous variable in the model. All the statistical tests were conducted using IBM SPSS version 26, GraphPad Prism version 9.4.0, and MedCalc version 20.015. A two-sided *P* value <  0.05 was considered statistically significant.

## 3. Results

### 3.1. Study population

We screened 854 patients who underwent AF ablation procedures involving RFA or CBA at our center between 2017 and 2021. We excluded 600 patients who did not have HF (n =  210), presented with non-paroxysmal AF (n =  162), had HFrEF or heart failure with mildly reduced ejection fraction (HFmrEF) (n =  186), underwent redo ablation (n =  39), or lacked essential baseline data (n =  3). Of the remaining 254 patients, 12 were ineligible due to complications such as severe valvular heart disease (n =  7), acute myocardial infarction (n =  3), acute HF (n =  1), or malignancies (n =  1). Ultimately, 242 patients met the inclusion criteria and were enrolled. During the one-year follow-up period, 38 patients were lost to follow-up, with one death unrelated to cardiovascular events, resulting in a dropout rate of 15.7%. Fig 1 shows the flowchart of the study.

Among the 204 patients included in this study, 43 (21.1%) were classified into the AF recurrence group, while 161 (78.9%) were classified into the non-AF recurrence group. Of these, 110 (53.9%) were male. The median age was 64.0 (57.0–70.0) years, with a mean BMI of 24.1 ±  2.9 kg/m^2^. The median LC, TC, and ALB were 1.6 (1.3–1.9)/mm^3^, 4.7 (4.6–4.8) mmol/L, and 42.2 (40.3–43.7) g/L, respectively. The mean NT-pro BNP was 593.7 ±  331.9 pg/mL. Significant differences were observed between the non-AF recurrence and recurrence groups in TC, ALB, and NT-pro BNP: [4.7 (4.7–4.8) mmol/L vs. 4.3 (4.3–4.4) mmol/L, *P* <  0.001], [42.9 (40.6–43.8) g/L vs. 40.5 (39.7–41.1) g/L, *P* <  0.001], and [485.4 ±  254.1 pg/mL vs. 999.3 ±  270.2 pg/mL, *P* <  0.001], respectively. Additionally, amiodarone and NOACs were the most commonly prescribed preprocedural medications, accounting for 96.6% and 86.8%, respectively, with 131 patients (64.2%) receiving statin therapy. Detailed baseline characteristics of the enrolled patients can be found in Table 2.

**Table 2:** Baseline characteristics of the patients.

Variables	All	Non-recurrence	Recurrence	*P* value
	N = 204	N = 161	N = 43	
**Demographics**				
Age (y)	64.0 (57.0–70.0)	64.0 (57.0–69.0)	64.0 (54.5–70.0)	0.832
Male (%)	110 (53.9)	83 (51.6)	27 (62.8)	0.189
**Basic information**				
SBP (mmHg)	134.4 ± 18.2	134.9 ± 18.4	132.9 ± 17.3	0.529
DBP (mmHg)	79.4 ± 11.0	79.4 ± 10.7	79.1 ± 12.3	0.854
heart rate (beats/min)	72.9 ± 12.2	73.2 ± 12.4	71.6 ± 11.5	0.438
BMI (kg/m^2^)	24.1 ± 2.9	24.1 ± 2.9	23.9 ± 2.7	0.658
CHA_2_DS_2_-VASc	2.0 (1.0 –3.3)	2.0 (1.0–4.0)	2.0 (1.0–3.0)	0.690
HAS-BLED	1.0 (0–2.0)	1.0 (0–2.0)	1.0 (0–2.0)	0.592
KCCQ score	81.2 (75.0–81.2)	81.2 (75.0–81.2)	75 (56.7–81.2)	0.511
MLHFQ score	3.0 (3.0–3.5)	3.0 (3.0–3.0)	3.0 (2.0–26.0)	0.896
NYHA grade (n, %)				**0.022**
I	8 (3.9)	5 (3.1)	3 (7.0)	
II	167 (81.9)	138 (85.7)	29 (67.4)	
III	29 (14.2)	18 (11.2)	11 (25.6)	
**Comorbidities**				
Hypertension (n, %)	124 (60.8)	101 (62.7)	23 (53.5)	0.270
Diabetes (n, %)	29 (14.2)	27 (16.8)	2 (4.7)	**0.043**
CHD (n, %)	61 (29.9)	48 (29.8)	13 (30.2)	0.957
Stroke (n, %)	32 (15.7)	24 (14.9)	8 (18.6)	0.554
COPD (n, %)	8 (3.9)	6 (3.7)	2 (4.7)	0.980
**Laboratory data**				
LC (/mm^3^)	1.6 (1.3–1.9)	1.6 (1.2–1.9)	1.5 (1.3–1.8)	0.941
eGFR (ml/min/1.73m^2^)	84.6 ± 16.9	84.1 ± 17.2	86.6 ± 15.9	0.404
Uric acid (umol/L)	335.3 ± 86.1	333.5 ± 87.7	342.1 ± 80.5	0.561
TC (mmol/L)	4.7 (4.6–4.8)	4.7 (4.7–4.8)	4.3 (4.3–4.4)	** < 0.001**
ALB (g/L)	42.2 (40.3–43.7)	42.9 (40.6–43.8)	40.5 (39.7–41.1)	** < 0.001**
NT-pro BNP (pg/mL)	593.7 ± 331.9	485.4 ± 254.1	999.3 ± 270.2	** < 0.001**
**Parameters of TEE**				
LA diameter (mm)	36.4 ± 4.4	36.3 ± 4.6	36.9 ± 3.9	0.405
RA diameter (mm)	32.8 ± 4.3	32.7 ± 4.2	33.4 ± 4.7	0.315
LVEF (%)	61.2 ± 4.6	61.0 ± 4.2	61.8 ± 6.1	0.420
**Types of procedure**				0.785
RFA	77 (37.7)	60 (37.3)	17 (39.5)	
CBA	127 (62.3)	101 (62.7)	26 (60.5)	
**Prior medications**				
β blocker (n, %)	116 (56.9)	94 (58.4)	22 (51.2)	0.396
Amiodarone (n, %)	197 (96.6)	155 (96.3)	42 (97.7)	0.654
Warfarin (n, %)	41(20.1)	28 (17.4)	13 (30.2)	0.062
NOACs (n, %)	177 (86.8)	142 (88.2)	35 (81.4)	0.242
Statin (n, %)	131 (64.2)	104 (64.6)	27 (62.8)	0.826
**Nutritional indices**				
CONUT	1 (0–2)	1 (0–2)	2 (1–2)	** < 0.001**
High CONUT	148 (72.5)	105 (65.2)	40 (93.0)	
Low CONUT	56 (27.5)	56 (34.8)	3 (7.0)	
NRI	104.5 (102.2–107.8)	105.8 (102.4–108.1)	102.8 (100.9–103.8)	** < 0.001**
High NRI	97 (47.5)	93 (57.8)	4 (9.3)	
Low NRI	107 (52.5)	68 (42.2)	39 (90.7)	
PNI	49.7 (47.7–52.3)	50.6 (48.0–52.6)	48.2 (46.9–49.9)	** < 0.001**
High PNI	89 (43.6)	82 (50.9)	7 (16.3)	
Low PNI	115 (56.4)	79 (49.1)	36 (83.7)	

The values are presented as the mean ±  standard deviation, median (interquartile range) or n (%). A *P* value <  0.05 indicated statistical significance.

Abbreviations: ALB, albumin; BMI, body mass index; CBA, cryoballoon ablation; CHA_2_DS_2_-VASc, congestive heart failure, hypertension, age ≥  75 years, diabetes mellitus, stroke, vascular disease, age 65–74 years, sex category; CHD, coronary heart disease; CONUT, controlling nutritional status; COPD, chronic obstructive pulmonary disease; DBP, diastolic blood pressure; eGFR, estimated glomerular filtration rate; HAS-BLED, hypertension, abnormal renal/hepatic function, stroke, bleeding history or predisposition, labile normalized ratio, elderly, drugs/alcohol concomitantly; KCCQ, kansas city cardiomyopathy questionnaire; LA, left atrial; LC, lymphocyte count; LVEF, left ventricular ejection fraction; MLHFQ, minnesota living with heart failure questionnaire; NOACs, novel oral anticoagulants; NRI, nutritional risk index; NT-pro BNP, N-terminal pro-brain natriuretic peptide; NYHA, New York heart association; PNI, prognostic nutritional index; RA, right atrial; RFA, radiofrequency ablation; SBP, systolic blood pressure; TC, total cholesterol; TEE, transesophageal echocardiography.

### 3.2. Prevalence of nutritional risk

Significant differences were observed in the CONUT score, NRI, or PNI between the AF recurrence group and non-AF recurrence groups, while there was no significant difference in body mass index (BMI). The median CONUT score was 1 (0–2), the median NRI was 104.5 (102.2–107.8), and the median PNI was 49.7 (47.7–52.3) (Table 2). Based on the CONUT score and NRI, 81 (39.7%) and 11 (5.4%) patients were identified as having a mild nutritional risk. According to the NRI, 5 (2.5%) patients were categorized as having a moderate risk of malnutrition (S1 Table). When considering BMI, the highest incidence of nutritional risk was observed among patients classified as underweight or with a normal weight by the CONUT score (40.0%) and NRI (13.3%). Notably, a substantial proportion of patients with BMI of an overweight or obese were still at risk for malnutrition, as indicated by the CONUT score (39.4%) and NRI (2.0%) (S2 Table).

### 3.3. Nutritional risk and AF recurrence

**Fig 2:**
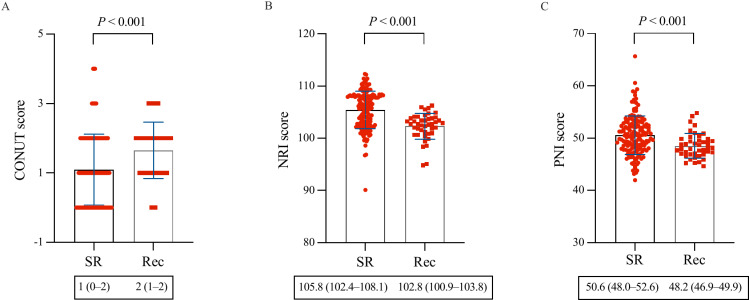
Comparison of baseline nutritional status between the AF recurrence group and the non-AF recurrence group. AF, atrial fibrillation; CONUT, controlling nutritional status; NRI, nutritional risk index; PNI, prognostic nutritional index; Rec, recurrence; SR, sinus rhythm.

**Fig 3:**
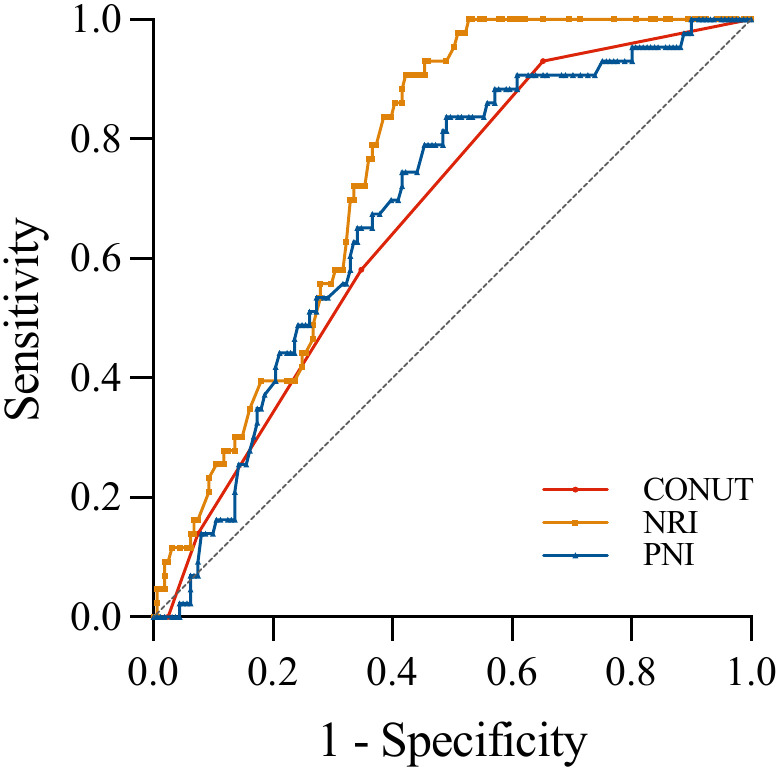
ROC curves of the CONUT score, NRI, and PNI for predicting recurrence of AF. AF, atrial fibrillation; CONUT, controlling nutritional status; NRI, nutritional risk index; PNI, prognostic nutritional index; ROC, receiver operating characteristic.

During a mean follow-up period of 11.2 ±  1.8 months, 43 patients (21.1%) experienced AF recurrence. Comparing the non-AF recurrence and AF recurrence groups, the CONUT score, NRI, or PNI exhibited significant differences (1 vs. 2, *P* <  0.001; 105.8 vs. 102.8, *P* <  0.001; 50.6 vs. 48.2, *P* <  0.001) (Fig 2). ROC curves were constructed for the CONUT score, NRI, and PNI to assess the prognostic value of AF recurrence (Fig 3). The AUCs of the CONUT score, NRI, and PNI were 0.668 (95% CI: 0.599–0.732, *P* <  0.001), 0.752 (95% CI: 0.687–0.810, *P* <  0.001), and 0.682 (95% CI: 0.613–0.745, *P* <  0.001), respectively. However, there were no significant differences concerning AUC among the nutritional screening tools (Table 3). Based on the ROC curve-derived optimal values of 0.5 for the CONUT score, 104.9 for the NRI, and 50.45 for the PNI, patients were categorized into two groups to predict AF recurrence following ablation, as shown in Table 4.

**Table 3:** The areas under the ROC curves and cutoff values of nutritional screening tools for AF recurrence.

Variables	AUC	95% CI	*P* value	Cutoff value
CONUT	0.668	0.599–0.732	** < 0.001**	0.5
NRI	0.752	0.687–0.810	** < 0.001**	104.9
PNI	0.682	0.613–0.745	** < 0.001**	50.45
**Variables**	**Differences between areas**	**95% CI**	***P* value**	
CONUT-NRI	-0.0848	-0.0193–0.1890	0.1104	
CONUT-PNI	-0.0142	-0.0588–0.0872	0.7024	
NRI-PNI	0.0706	-0.0157–0.1570	0.1087	

A *P* value <  0.05 indicated statistical significance.

Abbreviations: AF, atrial fibrillation; AUC, area under the curve; CI, confidence interval; CONUT, controlling nutritional status; NRI, nutritional risk index; PNI, prognostic nutritional index; ROC, receiver operating characteristic.

**Table 4:** Cox regressions of the relationships between three nutritional screening tools and the risk of AF recurrence.

Variables	Unadjusted			Adjusted		
	HR	95% CI	*P* value	HR	95% CI	*P* value
CONUT, continuous	1.536	1.170–2.016	**0.002**	1.701	1.143–2.532	**0.009**
CONUT, categorical						
Low CONUT	Ref	Ref	Ref	Ref	Ref	Ref
High CONUT	6.045	1.870–19.546	**0.003**	10.132	2.545–40.336	**0.001**
NRI, continuous	0.831	0.778–0.887	** < 0.001**	0.682	0.593–0.783	** < 0.001**
NRI, categorial						
High NRI	Ref	Ref	Ref	Ref	Ref	Ref
Low NRI	10.760	3.843–30.129	** < 0.001**	22.734	6.399–80.776	** < 0.001**
PNI, continuous	0.847	0.768–0.933	**0.001**	0.785	0.673–0.915	**0.002**
PNI, categorial						
High PNI	Ref	Ref	Ref	Ref	Ref	Ref
Low PNI	4.401	1.958–9.892	** < 0.001**	9.469	3.232–27.739	** < 0.001**

A *P* value <  0.05 indicated statistical significance.

The following variables were adjusted for: sex, age, BMI, SBP, DBP, heart rate, hypertension, diabetes, stroke, coronary artery disease, COPD, NYHA grade, KCCQ score, MLHFQ score, CHA_2_DS_2_-VASc score, NT-pro BNP, LA, RA, LVEF, type of ablation procedure, and eGFR.

Abbreviations: AF, atrial fibrillation; BMI, body mass index; CHA_2_DS_2_-VASc, congestive heart failure, hypertension, age ≥  75 years, diabetes mellitus, stroke, vascular disease, age 65–74 years, sex category; CI, confidence interval; CONUT, controlling nutritional status; COPD, chronic obstructive pulmonary disease; DBP, diastolic blood pressure; HR, hazard ratio; eGFR, estimated glomerular filtration rate; HR, hazard ratio; KCCQ, kansas city cardiomyopathy questionnaire; LA, left atrial; LVEF, left ventricular ejection fraction; MLHFQ, minnesota living with heart failure questionnaire; NRI, nutritional risk index; NT-pro BNP, N-terminal pro-brain natriuretic peptide; NYHA, New York heart association; PNI, prognostic nutritional index; RA, right atrial; Ref, reference; SBP, systolic blood pressure.

**Fig 4:**
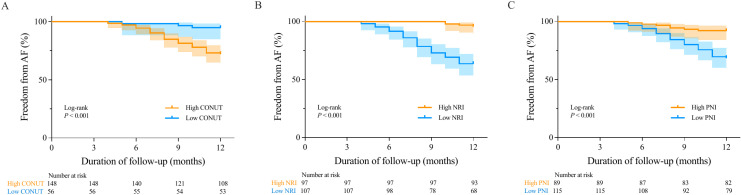
Kaplan–Meier survival curves of AF recurrence according to the CONUT (A), NRI (B), and PNI (C) scoring systems. AF, atrial fibrillation; CONUT, controlling nutritional status; NRI, nutritional risk index; PNI, prognostic nutritional index.

Kaplan–Meier curves revealed that patients with a high nutritional risk, as assessed by the CONUT score, NRI, and PNI, had significantly greater AF recurrence rates than those with low nutritional risk (log-rank *P* <  0.001) (Fig 4). In the univariate Cox regression analyses, predictors of AF recurrence included N-terminal prohormone of brain natriuretic peptide (NT-pro BNP), TC, and serum ALB (S3 Table), and patients with high CONUT score (HR: 6.045, 95% CI: 1.870–19.546, *P* =  0.003), low NRI (HR: 10.760, 95% CI: 3.843–30.129, *P* <  0.001), and low PNI (HR: 4.401, 95% CI: 1.958–9.892, *P* <  0.001) showed significantly higher AF recurrence rates (Table 4). After adjusting for patients’ demographic characteristics, symptom burden, laboratory results, type of ablation procedure, and echocardiographic measurements, the multivariate Cox model demonstrated a significant increase in AF recurrence rates for patients with high CONUT score (HR: 10.132, 95% CI: 2.545–40.336, *P* =  0.001), low NRI (HR: 22.734, 95% CI: 6.399–80.776, *P* <  0.001), and low PNI (HR: 9.469, 95% CI: 3.232–27.739, *P* <  0.001) (Table 4). The results of the Cox regression analysis with the CONUT score, NRI, and PNI as continuous variables indicated a statistically significant association between an increase in the CONUT score (HR: 1.701, 95% CI: 1.143–2.532, *P* =  0.009), a decrease in the NRI (HR: 0.682, 95% CI: 0.593–0.783, *P* <  0.001), and a decrease in the PNI (HR: 0.785, 95% CI: 0.673–0.915, *P* =  0.002) and an increased risk of AF recurrence (Table 4 and S1 Fig). Furthermore, in the sensitivity analysis, a consistent trend was observed (CONUT score: *P* for trend =  0.001, NRI: *P* for trend <  0.001, and PNI: *P* for trend =  0.001) (Table 5).

**Table 5:** Cox regression and trend analysis of three nutritional screening tools based on quartiles.

Variables	Median	Cases	Unadjusted				Adjusted			
			HR	95% CI	*P* value	*P* for trend	HR	95% CI	*P* value	*P* for trend
CONUT										
Q1 (≤0)	0	59	Ref	Ref	Ref		Ref	Ref	Ref	
Q2 (0–1]	1	64	5.038	1.458–17.403	0.011		8.255	1.897–35.922	0.005	
Q3 (1–2]	2	63	6.684	1.977–22.593	0.002		12.005	2.856–50.472	0.001	
Q4 (>2)	3	18	7.545	1.886–30.179	0.004	**0.001**	14.191	2.473–81.454	0.003	**0.001**
NRI										
Q1 (≤102.16)	100.94	53	Ref	Ref	Ref		Ref	Ref	Ref	
Q2 (102.16–104.51]	103.22	49	1.129	0.591–2.156	0.714		0.613	0.269–1.399	0.245	
Q3 (104.51–107.78]	106.28	53	0.272	0.107–0.690	0.006		0.086	0.026–0.278	< 0.001	
Q4 (>107.78)	108.99	49	0.000	–	0.928	** < 0.001**	0.000	–	0.919	** < 0.001**
PNI										
Q1 (≤47.66)	46.35	51	Ref	Ref	Ref		Ref	Ref	Ref	
Q2 (47.66–49.70]	48.80	52	0.640	0.318–1.287	0.210		0.378	0.150–0.955	0.040	
Q3 (49.70–52.30]	50.90	51	0.333	0.139–0.798	0.014		0.092	0.027–0.311	< 0.001	
Q4 (>52.30)	53.83	50	0.188	0.064–0.556	0.003	** < 0.001**	0.267	0.080–0.888	0.031	**0.001**

A P value < 0.05 indicated a statistically significant difference.

The following variables were adjusted for: sex, age, BMI, SBP, DBP, heart rate, hypertension, diabetes, stroke, coronary artery disease, COPD, NYHA grade, KCCQ score, MLHFQ score, CHA_2_DS_2_-VASc score, NT-pro BNP, LA, RA, LVEF, type of ablation procedure, and eGFR.

Abbreviations: BMI, body mass index; CHA_2_DS_2_-VASc, congestive heart failure, hypertension, age ≥  75 years, diabetes mellitus, stroke, vascular disease, age 65–74 years, sex category; CI, confidence interval; CONUT, controlling nutritional status; COPD, chronic obstructive pulmonary disease; DBP, diastolic blood pressure; eGFR, estimated glomerular filtration rate; HR, hazard ratio; KCCQ, kansas city cardiomyopathy questionnaire; LA, left atrial; LVEF, left ventricular ejection fraction; MLHFQ, minnesota living with heart failure questionnaire; NRI, nutritional risk index; NT-pro BNP, N-terminal pro-brain natriuretic peptide; NYHA, New York heart association; PNI, prognostic nutritional index; RA, right atrial; Ref, reference; SBP, systolic blood pressure.

A worsening nutritional status, as assessed by the CONUT score and NRI, was associated with a greater incidence of AF recurrence. Compared to patients with a normal nutritional status, those with mild nutritional risk assessed by the CONUT score had an increased risk of AF recurrence (multivariate HR: 2.916, 95% CI: 1.285–6.617, *P* =  0.010) (S4 Table).

**Fig 5:**
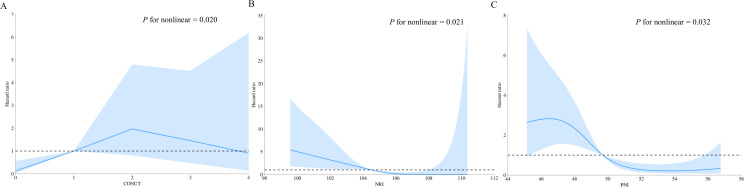
Restricted cubic spline regression for the associations between three nutritional screening tools and AF recurrence. Blue lines represent the HR, and blue transparent areas represent the 95% CI. (A) Association between CONUT score and AF recurrence; (B) Association between NRI and AF recurrence; (C) Association between PNI and AF recurrence. AF, atrial fibrillation; CI, confidence interval; CONUT, controlling nutritional status; HR, hazard ratio; NRI, nutritional risk index; PNI, prognostic nutritional index.

In restricted cubic spline regression, the CONUT score, NRI, and PNI all showed significant non-linear relationships with AF recurrence post-ablation (non-linear *P* value <  0.05). An inverted U-shaped relationship was observed between the HR of AF recurrence and the CONUT score in the overall population (Fig 5A). A higher CONUT score indicates poorer nutritional status. For the CONUT score, the HR increased gradually until it reached approximately 2, after which there was a downward trend. The HR of AF recurrence and NRI showed an L-shaped relationship (Fig 5B). For NRI, the HR decreased sharply to around 104–105; thereafter, the HR tended to decrease slowly. Additionally, the HR of AF recurrence and PNI displayed an L-shaped relationship (Fig 5C). For PNI, the HR gradually decreased to 49–50, and then the downward trend leveled off.

## 4. Discussion

This retrospective study investigated the impact of nutritional risk on AF recurrence after catheter ablation in patients with paroxysmal non-valvular AF and HFpEF. The key findings were as follows: (i) nutritional risk is prevalent in this population, with some patients classified as overweight or obese based on BMI still showing nutritional risk according to the CONUT score, NRI, and PNI; (ii) nutritional risk, assessed by the CONUT score, NRI, or PNI, is an independent risk factor for AF recurrence; and (iii) the HR for AF recurrence exhibits an L-shaped relationship with both the NRI and PNI, while the relationship with the CONUT score follows an inverted U-shape.

Malnutrition is a prevalent but often underestimated issue in clinical practice, particularly in patients with cardiovascular disease, where it can exacerbate the decline in cardiac function. Early identification and correction of malnutrition are crucial to improving patient quality of life and reducing cardiovascular-related risks. In this study, we employed three objective nutritional screening tools—CONUT score, NRI, and PNI—which minimize the influence of subjective factors and offer a standardized approach to assess nutritional status [13–15]. These tools are particularly beneficial for critically ill cardiovascular patients, who may struggle to provide reliable subjective feedback. Previous studies have demonstrated that nutritional risk, assessed by the CONUT score, NRI, or PNI, is associated with adverse outcomes in cardiovascular conditions such as myocardial infarction, HF, and stroke [26–28]. Furthermore, a recent study revealed an association between malnutrition and an increased risk of AF recurrence after catheter ablation in patients with paroxysmal AF [21]. Notably, HFpEF frequently coexists with AF, contributing to a poor prognosis. Although catheter ablation has been shown to improve the quality of life in patients with AF and HFpEF [29], the relationship between malnutrition and AF recurrence in this specific population has not been extensively studied. For the first time, we explored the relationship between nutritional risk and AF recurrence post-ablation in patients with paroxysmal non-valvular AF and HFpEF. This study provides valuable insights that may help may help improve the long-term prognosis of these patients. The inclusion of three widely used and validated nutritional screening tools—CONUT score, NRI, and PNI—offers a more comprehensive assessment of nutritional risk, allowing for a more reliable prediction of AF recurrence compared to BMI. Additionally, the study design carefully adjusted for multiple confounders, enhancing the robustness of the findings. These results have clinical relevance, suggesting potential strategies for personalized management in the post-ablation setting to optimize long-term outcomes.

The prevalence of nutritional risk in patients varies depending on the assessment system used [17]. While BMI is a commonly used indicator for assessing nutritional risk, our research revealed that even among patients classified as overweight or obese based on BMI, a portion of patients were identified as malnourished according to the CONUT score, NRI and PNI. In studies concerning AF recurrence post-ablation among patients with HFpEF, malnourished patients are often excluded from the study population, with BMI commonly utilized as a measure of nutritional risk assessment [29–31]. However, this approach might overlook the role of nutritional risk in AF recurrence post-ablation. BMI has limitations when applied to patients with AF. Although research has indicated that a BMI <  18.5 kg/m^2^ is a risk factor for AF recurrence [32], study by Bunch et al. [33] found that AF recurrence rates significantly increase with higher BMI. AF often results in multiple comorbidities, which can greatly influence a patient’s BMI [34]. During the compensation phase for certain diseases, such as HFpEF and diabetes, patients may become overweight or obese, leading to higher BMIs. Conversely, during the decompensation phase of some diseases, such as renal failure or end-stage HF, malnutrition due to cachexia can lead to a significant decrease in BMI. Furthermore, BMI has limitations in distinguishing between fat, muscle, and bone mass, potentially resulting in significant differences in body composition and metabolic status among patients with similar BMI values [21]. In contrast to BMI, the PNI evaluates an individual’s protein reserves and immune defense capabilities. The CONUT score offers a more comprehensive assessment of protein reserves, energy expenditure, and immune defense, while the NRI considers the combined impact of protein stores and body weight. Therefore, BMI alone may not accurately reflect the nutritional status of patients with AF and HFpEF. Utilizing the CONUT score, NRI or PNI can more effectively assess nutritional risk in these patients.

The ongoing debate regarding the superiority of the CONUT score and NRI in patients with HF has drawn attention. Our study revealed the effectiveness of the CONUT score, NRI, and PNI as predictors of AF recurrence in patients with paroxysmal non-valvular AF and HFpEF. While the NRI is constrained by protein reserves and body weight, it remains a potent independent predictor of HF mortality [35]. There is a notable correlation between HFpEF and obesity, often accompanied by hyperlipidemia. Patients with HFpEF may exhibit sarcopenic obesity, characterized by elevated BMI, low muscle mass, multiple comorbidities, excessive visceral adiposity, and heightened systemic inflammation [36]. Notably, TC concentration, one of the vital components of the CONUT score, can be influenced by the frequency of dyslipidemia and the use of statin medications [37]. In our study, despite only 29.9% of patients having coronary heart disease, statins usage was as high as 64.2%. Therefore, the NRI may be better suited than the CONUT for predicting AF recurrence in patients with AF and HFpEF who are taking statins.

After adjusting for confounding factors, we found that nutritional risk based on the CONUT score, NRI, or PNI was an independent risk factor for AF recurrence in patients with paroxysmal non-valvular AF and HFpEF. Furthermore, the AF recurrence rate in patients at risk of malnutrition was significantly greater than that in patients without malnutrition risk, aligning with previous research findings [19,21]. Malnutrition in patients with HFpEF represents a highly detrimental condition termed ‘cardiac cachexia’, serving as a potent predictor of all-cause mortality. This condition disrupts protein and heat balance, activates proinflammatory cytokines and the immune system, and leads to neurohormonal dysfunction [38,39]. Inflammation plays a crucial role in the onset and progression of both HFpEF and AF. Experimental evidence has indicated that the nucleotide-binding oligomerization domain-like receptor protein 3 (NLRP3) inflammasome and mitochondrial dysfunction may serve as pivotal driving factors in the development of HFpEF [40]. The STEP-HFpEF study [41] (trial code NCT04788511) also demonstrated that the use of semaglutide can significantly reduce weight and improve inflammation status in obese patients with HFpEF. Metabolic conditions such as obesity and diabetes can activate the NLRP3 inflammasome and other inflammatory cytokines in atrial myocytes, contributing to atrial remodeling and AF promotion [42]. Adipokines such as adiponectin play a crucial role in anti-inflammatory processes [43], but patients with the risk of malnutrition experience a reduced body fat, weakening the protective role of adipokines. These findings underscore the importance of nutritional screening tools in evaluating and managing patients with HFpEF undergoing AF ablation. These tools not only aid in predicting the risk of AF recurrence but also hold potential value in improving patient outcomes and devising intervention strategies to improve long-term prognosis.

The restricted cubic spline showed an L-shaped relationship between nutritional screening tools (NRI and PNI) and AF recurrence, indicating that nutritional risk significantly increases the risk of AF recurrence post-ablation. However, for the CONUT score, the HR of AF recurrence exhibits an inverted U-shaped relationship. This may be related to the generally good nutritional status of patients undergoing AF ablation at our center. For patients with extremely poor nutritional status, such as critically ill or very elderly individuals, physicians often prefer conservative medical treatments over ablation due to concerns about procedural tolerance. Large prospective studies focusing on this special population are needed in the future to further validate the reliability of these findings.

Clinicians should incorporate nutritional risk assessment into the pre-ablation evaluation, particularly for patients with comorbidities such as HFpEF, which may predispose them to malnutrition. Patients identified as nutritional risk based on three nutritional screening tools should undergo careful observation and follow-up post-ablation. Tailored strategies, including extended antiarrhythmic drug therapy or secondary ablation, may be necessary to reduce the burden of AF. Additionally, early identification of nutritional risk should prompt efforts to uncover underlying causes and provide appropriate nutritional support, potentially improving long-term outcomes in patients with AF and HFpEF.

## 5. Study limitations

This study has several limitations: (i) It was a retrospective study conducted at a single center. The overall study population included patients scheduled for AF ablation therapy with relatively preserved nutritional status. Therefore, the results may not capture the characteristics of other patient groups, such as pregnant women or children. (ii) Nutritional status was assessed for most patients only once during the week preceding hospitalization, potentially overlooking changes during the post-ablation period and failing to accurately reflect patients’ nutritional status. (iii) The lack of continuous monitoring of AF recurrence in this study may have underestimated the actual AF recurrence rate among patients. (iv) The study did not include individuals from diverse racial backgrounds; therefore, the conclusions need further validation in different ethnic groups. (v) The relatively small sample size and specific patient population at a single center may limit the generalizability of the findings. Larger studies involving multiple centers and more diverse patient populations are needed to confirm the results. (vi) Some patients might have undetected underlying diseases contributing to nutritional risk. In the future, larger randomized controlled studies are needed to further clarify the relationship between nutritional status and AF recurrence in patients with AF and HFpEF.

## 6. Conclusion

In conclusion, this study underscores the importance of systematic nutritional assessment in patients with paroxysmal non-valvular AF and HFpEF undergoing catheter ablation. Nutritional risk assessed by the CONUT score, NRI, or PNI is prevalent in these patients, regardless of their BMI. Three nutritional screening tools effectively predict AF recurrence post-ablation in patients with paroxysmal AF and HFpEF. Furthermore, nutritional risk based on the CONUT score, NRI, or PNI is an independent risk factor for AF recurrence. These findings emphasize that patients at nutritional risk should undergo comprehensive evaluation and receive early appropriate nutritional support therapy to improve their long-term prognosis. Future large-scale randomized controlled trials are needed to elucidate the relationship between nutritional status and AF recurrence in this specific patient population.

## Supporting information

S1 Fig Forest plot showing the association between nutritional screening tools and the risk of AF recurrence with the CONUT score, NRI and PNI as continuous variables. (TIF)

S1 Table Baseline characteristics of nutritional risk levels according to nutritional screening tools. (DOCX)

S2 Table The relationship between BMI and nutritional risk levels based on three nutritional screening tools. (DOCX)

S3 Table The univariate Cox regressions of the relationships between baseline characteristics of the patients and the risk of AF recurrence. (DOCX)

S4 Table Cox regressions of the relationships between nutritional risk levels and the risk of AF recurrence. (DOCX)

S1 File Raw data. (CSV)

## Abbreviations

AF: atrial fibrillation
ALB: albumin
AUC: area under the curve
BMI: body mass index
CBA: cryoballoon ablation
CHD: coronary heart disease
CONUT: Controlling Nutritional Status
COPD: chronic obstructive pulmonary disease
DBP: diastolic blood pressure
ECG: electrocardiogram
eGFR: estimated glomerular filtration rate
HF: heart failure
HFmrEF: heart failure with mildly reduced ejection fraction
HFpEF: heart failure with preserved ejection fraction
HFrEF: heart failure with reduced ejection fraction
HR: hazard ratio
INR: international normalized ratio
KCCQ: Kansas City Cardiomyopathy Questionnaire
LC: lymphocyte count
LVEF: left ventricular ejection fraction
MLHFQ: Minnesota Living with Heart Failure Questionnaire
NLRP3: nucleotide-binding oligomerization domain-like receptor protein 3
NOACs: novel oral anticoagulants
NRI: Nutritional Risk Index
NT-pro BNP: N-terminal prohormone of brain natriuretic peptide
NYHA: New York Heart Association
PNI: Prognostic Nutritional Index
PVI: pulmonary vein isolation
RA: right atrial
RFA: radiofrequency ablation
ROC: receiver operating characteristic
TC: total cholesterol
TEE: transesophageal echocardiography.
