# Supplementary figures and images for: The risk of malnutrition as a predictor of arrhythmia recurrence after catheter ablation in patients with paroxysmal non-valvular atrial Fibrillation and heart failure with preserved ejection fraction
Source: PLoS One. 2025 Jan 31;20(1):e0317721. doi: 10.1371/journal.pone.0317721 (PMC11785320; doi:10.1371/journal.pone.0317721)

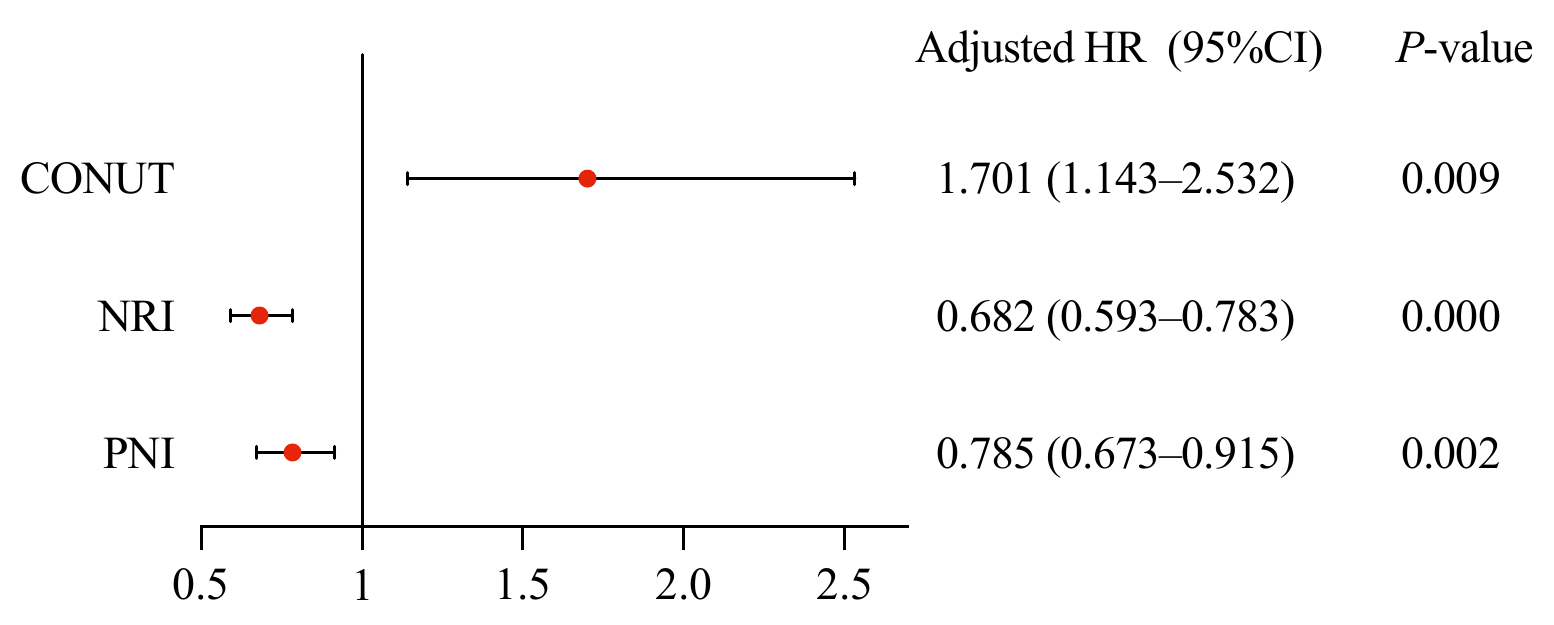

Supplement: S1 Fig — (TIF) [file pone.0317721.s001.tif]
