# Supplementary material for: The risk of malnutrition as a predictor of arrhythmia recurrence after catheter ablation in patients with paroxysmal non-valvular atrial Fibrillation and heart failure with preserved ejection fraction
Source: PLoS One. 2025 Jan 31;20(1):e0317721. doi: 10.1371/journal.pone.0317721 (PMC11785320; doi:10.1371/journal.pone.0317721)
Supplement: S1 Table — (DOCX) [file pone.0317721.s002.docx]

**S1 Table. Baseline characteristics of nutritional risk levels according to nutritional screening tools.**

| **Nutritional indexes** | **All** | **Non-recurrence** | **Recurrence** | ***P* value** |
| --- | --- | --- | --- | --- |
|  | N = 204 | N = 161 | N = 43 |  |
| **CONUT** | 1 (0–2) | 1 (0–2) | 2 (1–2) | **< 0.001** |
| Absent | 123 (60.3) | 105 (65.2) | 18 (41.9) |  |
| Mild | 81 (39.7) | 56 (34.8) | 25 (58.1) |  |
| **NRI** | 104.5 (102.2–107.8) | 105.8 (102.4–108.1) | 102.8 (100.9–103.8) | **< 0.001** |
| Absent | 188 (92.2) | 151 (93.8) | 37 (86.0) |  |
| Mild | 11 (5.4) | 7 (4.3) | 4 (9.3) |  |
| Moderate | 5 (2.5) | 3 (1.9) | 2 (4.7) |  |
| **PNI** | 49.7 (47.7–52.3) | 50.6 (48.0–52.6) | 48.2 (46.9–49.9) | **< 0.001** |
| Absent | 204 (100.0) | 161 (100.0) | 43 (100.0) |  |

The values are presented as the mean ± standard deviation, median (interquartile range) or n (%). A *P* value < 0.05 indicated statistical significance. AF, atrial fibrillation; CONUT, Controlling Nutritional Status; NRI, Nutritional Risk Index; PNI, Prognostic Nutritional Index.
