# Supplementary material for: The risk of malnutrition as a predictor of arrhythmia recurrence after catheter ablation in patients with paroxysmal non-valvular atrial Fibrillation and heart failure with preserved ejection fraction
Source: PLoS One. 2025 Jan 31;20(1):e0317721. doi: 10.1371/journal.pone.0317721 (PMC11785320; doi:10.1371/journal.pone.0317721)
Supplement: S2 Table — (DOCX) [file pone.0317721.s003.docx]

**S2 Table. The relationship between BMI and nutritional risk levels based on three nutritional screening tools.**

| **Nutritional indexes** | **All** | **BMI < 24** | **BMI ≥ 24** | ***P* value** |
| --- | --- | --- | --- | --- |
|  | N = 204 | N = 105 | N = 99 |  |
| **CONUT** | 1 (0–2) | 1 (0–2) | 1 (1–2) | 0.518 |
| Absent | 123 (60.3) | 63 (60.0) | 60 (60.6) | 0.930 |
| Mild | 81 (39.7) | 42 (40.0) | 39 (39.4) |  |
| **NRI** | 104.5 (102.2–107.8) | 103.8 (101.9–106.3) | 106.3 (102.6–108.1) | **0.001** |
| Absent | 188 (92.2) | 91 (86.7) | 97 (98.0) |  |
| Mild | 11 (5.4) | 9 (8.6) | 2 (2.0) |  |
| Moderate | 5 (2.5) | 5 (4.8) | 0 (0) |  |
| **PNI** | 49.7 (47.7–52.3) | 49.5 (47.7–52.3) | 49.7 (47.6–52.3) | 0.755 |
| Absent | 204 (100.0) | 105 (100.0) | 99 (100.0) |  |

The values are presented as the mean ± standard deviation, median (interquartile range) or n (%). A *P* value < 0.05 indicated statistical significance. Obesity is defined as BMI ≥ 28 kg/m^2^, while overweight is defined as 24 kg/m^2^ ≤ BMI < 28 kg/m^2^. BMI, body mass index; CONUT, Controlling Nutritional Status; NRI, Nutritional Risk Index; PNI, Prognostic Nutritional Index.
