# Supplementary material for: The risk of malnutrition as a predictor of arrhythmia recurrence after catheter ablation in patients with paroxysmal non-valvular atrial Fibrillation and heart failure with preserved ejection fraction
Source: PLoS One. 2025 Jan 31;20(1):e0317721. doi: 10.1371/journal.pone.0317721 (PMC11785320; doi:10.1371/journal.pone.0317721)
Supplement: S3 Table — (DOCX) [file pone.0317721.s004.docx]

**S3 Table. The univariate Cox regressions of the relationships between baseline characteristics of the patients and the risk of AF recurrence**

| **Variables** |  | **Unadjusted** | | |
| --- | --- | --- | --- | --- |
|  |  | HR | 95% CI | *P* value |
| **Demographics** |  |  | | |
| Age (y) |  | 0.995 | 0.964–1.028 | 0.769 |
| Male (%) |  | 0.656 | 0.354–1.218 | 0.182 |
| **Basic information** |  |  |  |  |
| SBP (mmHg) |  | 0.996 | 0.980–1.012 | 0.613 |
| DBP (mmHg) |  | 1.000 | 0.973–1.028 | 0.998 |
| Heart rate (beats/min) |  | 0.990 | 0.966–1.016 | 0.458 |
| BMI (kg/m^2^) |  | 0.969 | 0.871–1.078 | 0.561 |
| CHA_2_DS_2_-VASc |  | 0.947 | 0.804–1.115 | 0.511 |
| HAS-BLED |  | 0.921 | 0.700–1.212 | 0.556 |
| KCCQ score |  | 0.986 | 0.966–1.007 | 0.205 |
| MLHFQ score |  | 1.024 | 0.998–1.051 | 0.074 |
| NYHA grade (n, %) |  | 1.764 | 0.887–3.510 | 0.106 |
| **Comorbidities** |  |  | | |
| Hypertension (n, %) |  | 0.749 | 0.412–1.364 | 0.345 |
| Diabetes (n, %) |  | 0.267 | 0.065–1.106 | 0.069 |
| CHD (n, %) |  | 1.009 | 0.527–1.935 | 0.977 |
| Stroke (n, %) |  | 1.301 | 0.603–2.804 | 0.503 |
| COPD (n, %) |  | 1.306 | 0.316–5.401 | 0.712 |
| **Laboratory data** |  |  | | |
| LC (/mm^3^) |  | 1.015 | 0.581–1.774 | 0.959 |
| eGFR (ml/min/1.73m^2^) |  | 1.008 | 0.990–1.027 | 0.382 |
| Uric acid (umol/L) |  | 1.001 | 0.998–1.004 | 0.559 |
| TC (mg/dL) |  | 0.023 | 0.010–0.052 | **< 0.001** |
| ALB (g/dL) |  | 0.616 | 0.517–0.733 | **< 0.001** |
| NT-pro BNP (pg/mL) |  | 1.002 | 1.002–1.003 | **< 0.001** |
| **Parameters of TEE** |  |  | | |
| LA diameter (mm) |  | 1.029 | 0.962–1.100 | 0.410 |
| RA diameter (mm) |  | 1.032 | 0.968–1.100 | 0.337 |
| LVEF (%) |  | 1.045 | 0.975–1.119 | 0.211 |
| **Types of procedure** |  |  |  |  |
| RFA |  | Ref | Ref | Ref |
| CBA |  | 0.906 | 0.491–1.669 | 0.750 |
| **Prior medications** |  |  | | |
| AADs (n, %) |  | 1.605 | 0.221–11.661 | 0.640 |
| Warfarin (n, %) |  | 1.788 | 0.932–3.427 | 0.080 |
| NOACs (n, %) |  | 0.636 | 0.295–1.372 | 0.249 |

A *P* value < 0.05 indicated statistical significance. AADs, antiarrhythmic drugs; AF, atrial fibrillation; ALB, albumin; BMI, body mass index; CBA, cryoballoon ablation; CHA_2_DS_2_-VASc, congestive heart failure, hypertension, age ≥ 75 years, diabetes mellitus, stroke, vascular disease, age 65–74 years, sex category; CHD, coronary heart disease; CI, confidence interval; COPD, chronic obstructive pulmonary disease; DBP, diastolic blood pressure; eGFR, estimated glomerular filtration rate; HAS-BLED, hypertension, abnormal renal/hepatic function, stroke, bleeding history or predisposition, labile international normalized ratio, elderly, drugs/alcohol concomitantly; HR, hazard ratio; KCCQ, Kansas City Cardiomyopathy Questionnaire; LA, left atrial; LC, lymphocyte count; LVEF, left ventricular ejection fraction; MLHFQ, Minnesota Living with Heart Failure Questionnaire; NOACs, novel oral anticoagulants; NT-pro BNP, N-terminal pro-brain natriuretic peptide; NYHA, New York Heart Association; RA, right atrial; Ref, reference; RFA, radiofrequence ablation; SBP, systolic blood pressure; TC, total cholesterol; TEE, transesophageal echocardiography.
