# Supplementary material for: The risk of malnutrition as a predictor of arrhythmia recurrence after catheter ablation in patients with paroxysmal non-valvular atrial Fibrillation and heart failure with preserved ejection fraction
Source: PLoS One. 2025 Jan 31;20(1):e0317721. doi: 10.1371/journal.pone.0317721 (PMC11785320; doi:10.1371/journal.pone.0317721)
Supplement: S4 Table — (DOCX) [file pone.0317721.s005.docx]

**S4 Table. Cox regressions of the relationships between nutritional risk levels and the risk of AF recurrence.**

| **Variables** | **Unadjusted** | | |  | **Adjusted** | | |
| --- | --- | --- | --- | --- | --- | --- | --- |
|  | HR | 95% CI | *P* value |  | HR | 95% CI | *P* value |
| CONUT, continuous | 1.536 | 1.170–2.016 | **0.002** |  | 1.701 | 1.143–2.532 | **0.009** |
| CONUT, categorical |  |  |  |  |  |  |  |
| Absent | Ref | Ref | Ref |  | Ref | Ref | Ref |
| Mild | 2.281 | 1.244–4.182 | **0.008** |  | 2.916 | 1.285–6.617 | **0.010** |
| NRI, continuous | 0.831 | 0.778–0.887 | **< 0.001** |  | 0.682 | 0.593–0.783 | **< 0.001** |
| NRI, categorial |  |  |  |  |  |  |  |
| Absent | Ref | Ref | Ref |  | Ref | Ref | Ref |
| Mild | 2.453 | 0.874–6.886 | 0.088 |  | 2.966 | 0.823–10.685 | 0.096 |
| Moderate | 2.907 | 0.700–12.067 | 0.142 |  | 3.977 | 0.600–26.340 | 0.152 |
| PNI, continuous | 0.847 | 0.768–0.933 | **0.001** |  | 0.785 | 0.673–0.915 | **0.002** |

A *P* value < 0.05 indicated statistical significance. Adjust the following variables: sex, age, BMI, SBP, DBP, heart rate, hypertension, diabetes, stroke, coronary artery disease, COPD, NYHA grade, KCCQ score, MLHFQ score, CHA_2_DS_2_-VASc score, NT-pro BNP, LA, RA, LVEF, type of ablation procedure, and eGFR. AF, atrial fibrillation; BMI, body mass index; CHA_2_DS_2_-VASc, congestive heart failure, hypertension, age ≥ 75 years, diabetes mellitus, stroke, vascular disease, age 65–74 years, sex category; CI, confidence interval; CONUT, Controlling Nutritional Status; COPD, chronic obstructive pulmonary disease; DBP, diastolic blood pressure; eGFR, estimated glomerular filtration rate; HR, hazard ratio; KCCQ, Kansas City Cardiomyopathy Questionnaire; LA, left atrial; LVEF, left ventricular ejection fraction; MLHFQ, Minnesota Living with Heart Failure Questionnaire; NRI, Nutritional Risk Index; NT-pro BNP, N-terminal pro-brain natriuretic peptide; NYHA, New York Heart Association; PNI, Prognostic Nutritional Index; RA, right atrial; Ref, reference; SBP, systolic blood pressure.
